# Detection of a Novel Mechanism of Acousto-Optic Modulation of Incoherent Light

**DOI:** 10.1371/journal.pone.0104268

**Published:** 2014-08-08

**Authors:** Christopher W. Jarrett, Charles F. Caskey, John C. Gore

**Affiliations:** 1 Vanderbilt University Institute of Imaging Science, Vanderbilt University, Nashville, Tennessee, United States of America; 2 Program in Chemical and Physical Biology, Vanderbilt University, Nashville, Tennessee, United States of America; 3 Department of Radiology and Radiological Sciences, Vanderbilt University, Nashville, Tennessee, United States of America; 4 Department of Biomedical Engineering, Vanderbilt University, Nashville, Tennessee, United States of America; University of California Irvine, United States of America

## Abstract

A novel form of acoustic modulation of light from an incoherent source has been detected in water as well as in turbid media. We demonstrate that patterns of modulated light intensity appear to propagate as the optical shadow of the density variations caused by ultrasound within an illuminated ultrasonic focal zone. This pattern differs from previous reports of acousto-optical interactions that produce diffraction effects that rely on phase shifts and changes in light directions caused by the acoustic modulation. Moreover, previous studies of acousto-optic interactions have mainly reported the effects of sound on coherent light sources via photon tagging, and/or the production of diffraction phenomena from phase effects that give rise to discrete sidebands. We aimed to assess whether the effects of ultrasound modulation of the intensity of light from an incoherent light source could be detected directly, and how the acoustically modulated (AOM) light signal depended on experimental parameters. Our observations suggest that ultrasound at moderate intensities can induce sufficiently large density variations within a uniform medium to cause measurable modulation of the intensity of an incoherent light source by absorption. Light passing through a region of high intensity ultrasound then produces a pattern that is the projection of the density variations within the region of their interaction. The patterns exhibit distinct maxima and minima that are observed at locations much different from those predicted by Raman-Nath, Bragg, or other diffraction theory. The observed patterns scaled appropriately with the geometrical magnification and sound wavelength. We conclude that these observed patterns are simple projections of the ultrasound induced density changes which cause spatial and temporal variations of the optical absorption within the illuminated sound field. These effects potentially provide a novel method for visualizing sound fields and may assist the interpretation of other hybrid imaging methods.

## Introduction

We report novel experimental observations of the modulation of an incoherent light beam by an ultrasonic field that are distinct from previous reported interactions of sound and light, and which demonstrate the ability to directly observe sound pressure patterns as changes in light absorption. This work was originally motivated by our interest in developing and evaluating a novel type of hybrid imaging system for molecular imaging in biological samples, and in the interpretation of previous reports of acousto-optical imaging using fluorescent light sources. Optoacoustic/Photoacoustic imaging, in which sound is produced and detected after light interacts with a target, has been successfully developed as a hybrid imaging method that combines the molecular sensitivity of optical methods with the spatial resolution and depth penetration of ultrasound, and is now in practical use in clinical and pre-clinical applications [1]–[3]. Acousto-optical imaging is an alternative technology to photoacoustic imaging, in which sound is used to modulate a light source, but it potentially shares some of the advantages of photoacoustic imaging, and our studies were motivated by the need to better understand the nature of acousto-optic interactions. The ability of sound to modulate light by some means is well established, and has been extensively studied both theoretically and experimentally for many years. However, previous reports of effects of sound on light have described mainly diffraction phenomena caused by phase differences of light waves induced by sound fields, such as those by Brillouin [4], Raman and Nath [5]–[9], Debye and Sears [10], Bragg [11], Lucas and Biquard [12], Berry [13], and Wang [14], [15]. To our knowledge, there have been no previous reports of the direct effects of sound causing changes in the absorption of ballistic photons, as reported here.

Acousto-optical imaging was first studied in the early 1990s when Marks et al. experimented with the ability to “tag” light with ultrasound [16]. Wang used a related approach to acquire images of tissues phantoms, while Leutz and Maret theoretically and experimentally analyzed the ultrasonic modulation of light [17], [18]. To date, most reports of Acousto-Optical imaging have exploited the ultrasonic modulation only of coherent light to interrogate the optical properties of a region of interest. However, in order to detect the effects of ultrasound on the emissions from fluorescent sources within an optically turbid medium, it is necessary to be able to measure ultrasonic modulation of incoherent light. To date there have been only a small number of groups that have detected and reported the ultrasonic modulation of incoherent fluorescent light [19]–[23]. Kobayashi *et al*. were the first to develop an acousto-optical system to demonstrate the ability to ultrasonically modulate the fluorescent light and also image the distribution of embedded fluorophores tomographically [19], [20]. The mechanism behind their reported results is not well understood and to date, no other group has been successful in repeating their experiments using similar apparatus. In our own studies using a near-identical set up, we were unable to replicate the precise effects reported by Kobayashi et al., though we did observe robust modulation of the coherent light used to produce fluorescence and the consequent modulation of the emission from the fluorophores. This phenomenon has previously been termed the ultrasound tagging of photons [17], [18], [24]–[26] and is exploited in the techniques of ultrasound-modulated optical tomography (USMOT) also known as acousto-optical tomography (AOT) [27]. As a follow up to those studies we aimed to test whether acoustic modulation of incoherent light was detectable using reasonable sound intensities. To remove possible confounding effects of the modulation of any coherent light sources, instead of using a laser to excite a fluorophore we substituted an incoherent LED light source. This was similar to the experimental methods of Huyhn et al. in which a chemiluminescent source replaced the laser excited fluorophore [28]. The LED was used because it was easily controlled and characterized. Using this experimental setup, we were able to demonstrate and quantify the effects of ultrasound modulation on incoherent light, and here we report experimental evidence that the ultrasound focal zone produces a spatial variation of light absorption which, when projected, replicates the expected distribution of sound pressure and material density in the sound field. This effect differs from previous reports of diffraction phenomena caused by phase differences such as those mentioned above, and allows a much simpler approach to the observation of sound fields than those provided by previous work [29]–[32].

## Materials and Methods

**Figure 1:**
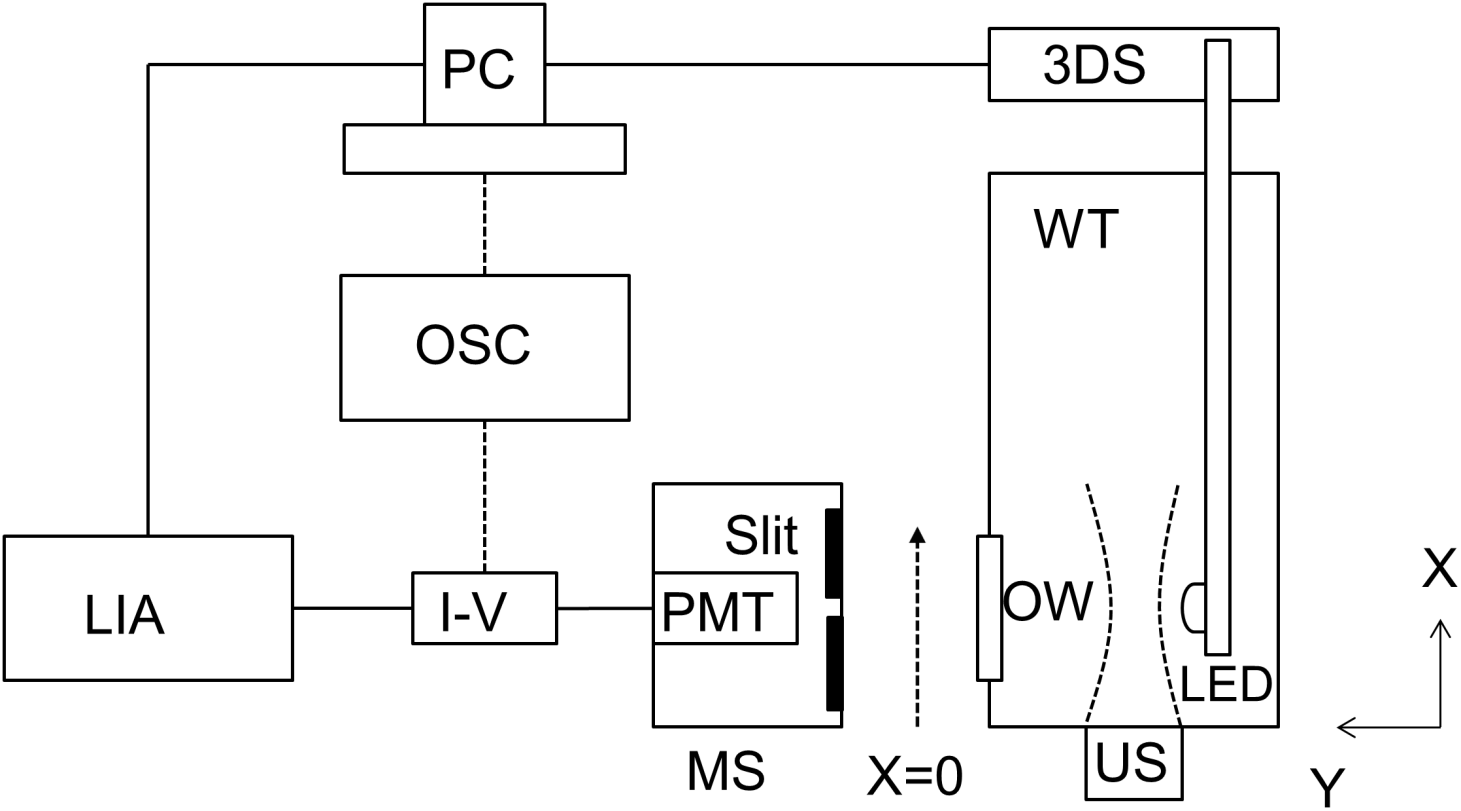
Experimental Apparatus. US: ultrasound transducer; LED: light-emitting diode; OW: optical window; WT: water tank; PMT: photomultiplier tube; MS: motion stage; I–V: transimpedance amplifier; LIA: lock-in amplifier; OSC: oscilloscope; 3DS: three axis motion stage; PC: LabVIEW system control and data acquisition computer.

Figure 1 shows the experimental system that was designed and built to be able to measure both optical and acousto-optical signals. A water tank was constructed with an opening for a transmitting ultrasound transducer (directed along the x-axis) and an orthogonal optical window (directed along the y-axis) centered at approximately 38.1 mm distance along the transducer (x) axis, and offset by 103 mm (along y). A waterproof LED light source (Super Bright LEDs, RL5-R8030, 630 nm) was attached to a three-dimensional translation stage and inserted into the water tank. The LED was positioned 10 mm along the y-axis from the ultrasound beam axis and directed towards the optical window. A focused circular ultrasound transducer (Olympus Panametrics V314, 1 MHz center frequency, 19.05 mm element size and 38.1 mm focal length, or a Valpey Fischer, IL0206HP, 2.25 MHz center frequency, 19.05 mm element size and 50.8 mm focal length) was placed such that the axial propagation of the ultrasonic beam (along x) was perpendicular to the principal direction (along y) of the LED light. The ultrasound focus of the 1 MHz transducer was located directly in front of the center of the optical window. A function generator (Agilent Technologies, 33500B) supplied a continuous wave, 1 MHz sinusoidal signal to an RF amplifier (Amplifier Research, 200 L) to drive the ultrasound transducer at a selected voltage (0–60 volts peak to peak) to achieve a corresponding ultrasound focal zone peak negative pressure of 0–60 kPa. The voltage to pressure conversion was calibrated and verified using a hydrophone (Onda HNC-0200). A photomultiplier tube (PMT) (Hamamatsu, H5783-20) and long, narrow sampling slit (0.75 mm width) were mounted to a translation stage at the optical window. The slit was positioned so that the light signal reaching the PMT was integrated vertically across the slit at the center of the PMT surface. The translation stages allowed the two dimensional movement of the PMT and slit to scan the pattern of LED light directed towards the PMT. The PMT signal was then passed through a trans-impedance amplifier (Hamamatsu, C6438) and amplified 20 dB and then input into a lock-in amplifier (Stanford Research Systems, SR844). The signal entering the lock-in amplifier was amplified a further 20 dB. The lock-in amplifier measured only the modulated light signal from the PMT that matches the frequency of the reference signal, which was the same as the ultrasound frequency. To record the entire incident light signal (modulated and unmodulated) reaching the PMT for a reference level, the output from the PMT could bypass the lock-in amplifier and be recorded directly on a recording oscilloscope (Hewlett Packard, 54503A). Both the lock-in amplifier and oscilloscope measured the signal amplitude (v) of the modulated input signal received from the PMT. However, we report all findings as the intensity of the modulated input signal (v^2^) or the squared signal amplitude. A computer with LabVIEW software was used to control all stage movements and data acquisition from the lock-in amplifier and/or the oscilloscope.

When the ultrasound transducer was excited, the PMT recorded an acousto-optic signal indicating there was a direct modulation of the light reaching the PMT at the transducer frequency. We first moved the PMT and slit in the x-direction (perpendicular to the principal direction of light propagation) to locate the position of the peak acousto-optical signal in an optically clear sample (water), and this was then recorded as a function of the ultrasound transducer voltage, which produced variations in the applied ultrasound peak pressure in the focal zone. The incident unmodulated light signal produced by the LED was also measured. The modulation depth, *M,* was then calculated at each ultrasound pressure using:
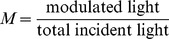
(1)


Next, we compared the acousto-optical modulated (AOM) signal after passing through an optically clear sample (water) and also within turbid media samples (0.125, 0.1825, and 0.25% volume, whole milk) while varying the applied ultrasound frequency (0.62, 1.0, 1.3, and 2.25 MHz) at a constant ultrasound pressure (∼60 kPa). The turbid media samples were not intended to be of physiological relevance as reduced scattering coefficients of dilute whole milk samples are expected in the mm^−1^ range [33], [34] while reduced scattering coefficients of biological tissues are expected in the cm^−1^ range [35], [36], but they were included to help isolate the mechanism of interaction. Two separate US transducers were used to achieve the desired frequencies, one with a center frequency of 1 MHz and the second with a center frequency of 2.25 MHz. Although the first ultrasound transducer was resonant at 1 MHz, the effect of the ultrasound on the LED light was measured by driving the transducer at 0.62, 1.0, and 1.3 MHz. At 0.62 and 1.3 MHz the transducer conversion efficiency was reduced by 50%, so we increased the driving voltage to compensate and verified the same peak pressure was achieved using the hydrophone. The spatial patterns of the AOM signals were measured by scanning the PMT and slit in 1 mm steps along the x-axis for each frequency at a fixed offset along y.

The overall geometry was also varied by adjusting the distance of the LED to the ultrasound focal zone between 10 and 16 mm respectively in 2 mm increments, and by varying the distance from the focal zone to the scanning slit from 103 to 130 mm. At each step in x, 100 data points were recorded and averaged. The spatial variation of the LED light reaching the PMT without any ultrasonic modulation was also measured.

## Results

**Figure 2:**
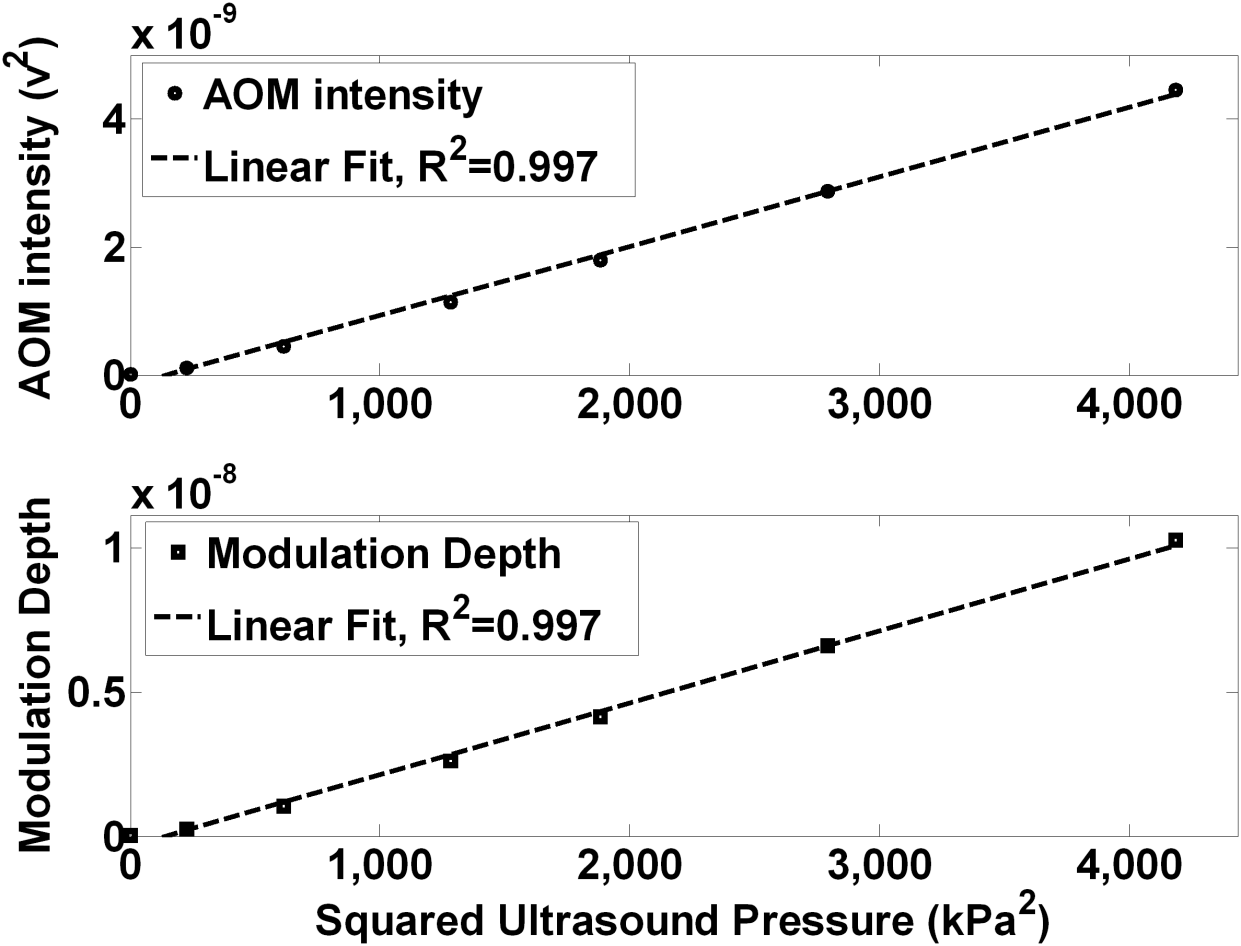
Acousto-optic modulation (AOM) intensity and modulation depth increase linearly with squared ultrasound pressure. For incoherent light traveling through an ultrasound focal zone, the AOM intensity (top) and modulation depth (bottom) are linearly proportional to the squared ultrasound pressure within the focal zone.

Figure 2 (top) shows the effect of the ultrasound pressure on the modulated light signal in water as measured by the lock-in amplifier. The AOM signal intensity was linearly proportional to the squared ultrasound pressure (R^2^ = 0.997). Figure 2 (bottom) shows that the modulation depth, M, was also linearly proportional to the squared ultrasound pressure (R^2^ = 0.997) with a peak modulation depth of ∼1×10^−8^ at an ultrasound pressure of ∼60 kPa.

**Figure 3:**
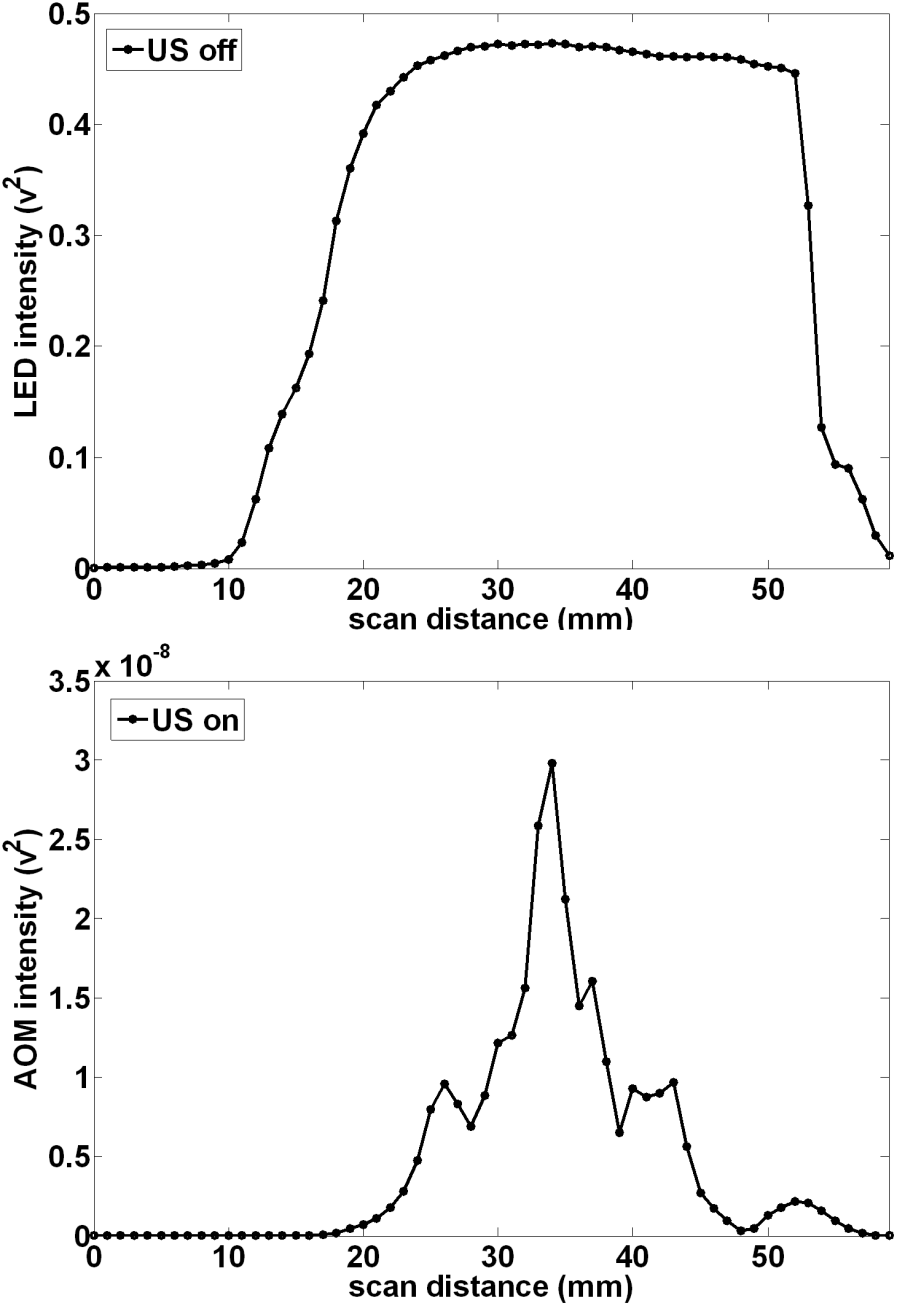
Ultrasound modulation causes a spatial pattern in the projection of incoherent LED light in water. When the unmodulated LED light propagation (RF off) is sampled at a projected distance of 113 mm, the normalized incoherent light distribution is relatively smooth and uniform over the detection window. However when the light passes through an ultrasound focal zone (1 MHz, located 10 mm from the LED and 103 mm from the projection plane), the light displays a pattern having a central peak with smaller maxima or side lobes on either side with an average peak spacing of 8.5 mm.

Figure 3 (top) shows the light distribution incident on the detector after passage through an optically clear medium (water) without ultrasound modulation, measured by scanning the PMT and slit with the LED to focal zone distance (d) = 10 mm and the LED to PMT projection distance (D) = 113 mm. The light pattern peaks about the principal axis of the LED at the center of the optical window, is reasonably uniform over approximately 30 mm of travel, but it then decreases monotonically as the slit moves further from the center of the LED and optical window. The limited size of the optical window and the geometry of the LED reduce the extent of the projection of the light at the plane of the detector. Figure 3 (bottom) shows the corresponding AOM signal pattern when the ultrasound is on. The overall pattern extends over approximately the same extent but there is a main narrow central peak with adjacent smaller maxima or side lobes on either side. The smaller maxima are located an average 8.5 mm from the main central peak. This pattern at first is suggestive of a far-field diffraction pattern, but as shown below, the pattern is in reality a simple projection of the variation of absorption of the light as it passes through the focal zone.

**Figure 4:**
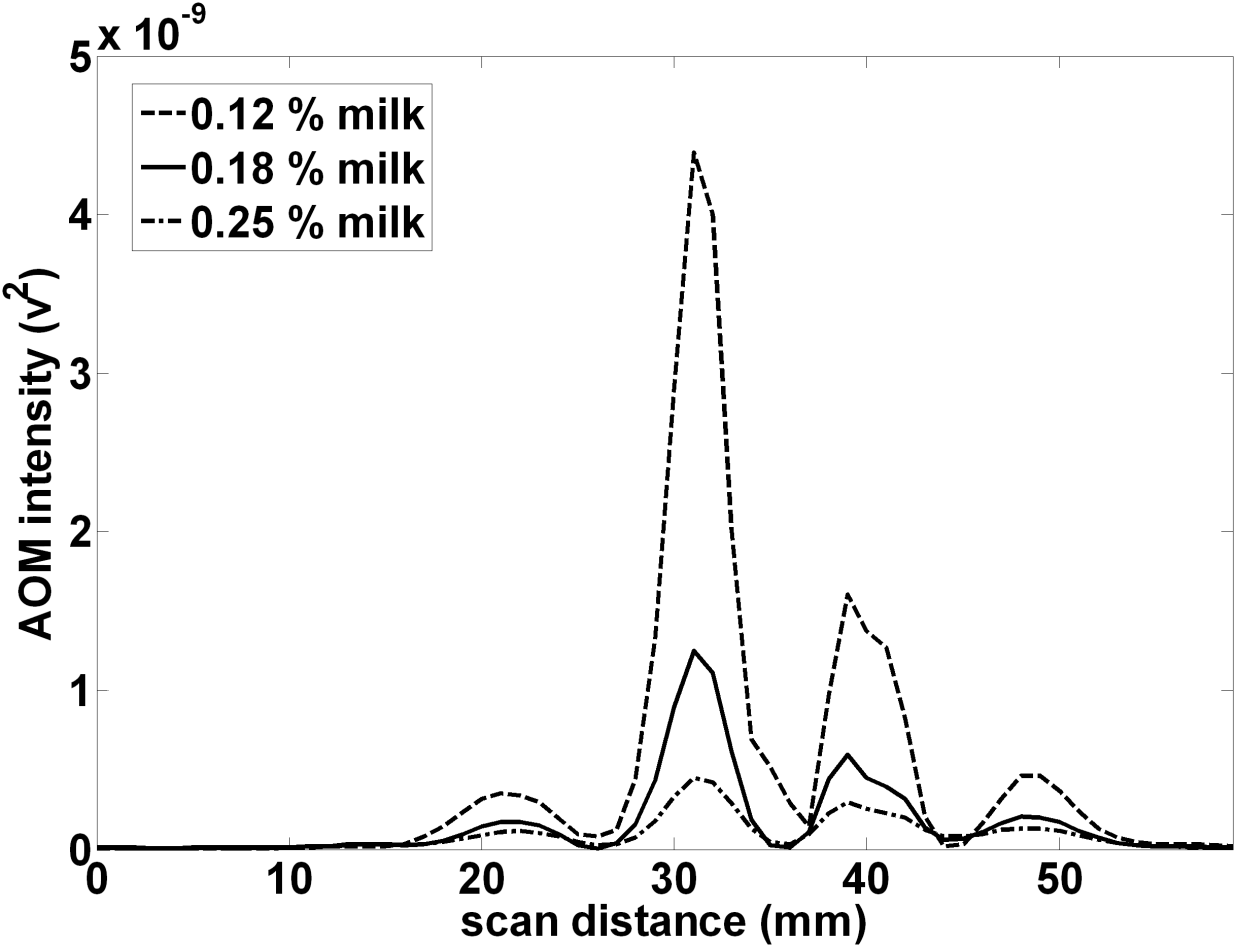
Ultrasound modulation causes a spatial pattern in the projection of incoherent LED light in milk. Passing LED light through a continuous wave ultrasound focal zone (1 MHz, located 10 mm from the LED and 103 mm from the projection plane) causes acoustic modulation of the light. In a turbid medium consisting of a suspension of milk, the projection of the LED light at 113 mm consists of a peak located at the center of the optical window and adjacent smaller maxima or side lobes. With increasing milk concentration, the spatial pattern does not change but the AOM signal decreases and the lobes appear better resolved.

Figure 4 shows the corresponding projection of the 1 MHz, acousto-optically modulated light after passage through a turbid media containing varying volume percentages of whole milk. Each projection displays a similar pattern with a main central peak and adjacent smaller maxima or side lobes. Increasing milk concentration decreases the peak AOM intensity, but the peaks in milk appear more clearly resolved as the minima are deeper. The average distance between peaks was measured to be approximately the same.

**Figure 5:**
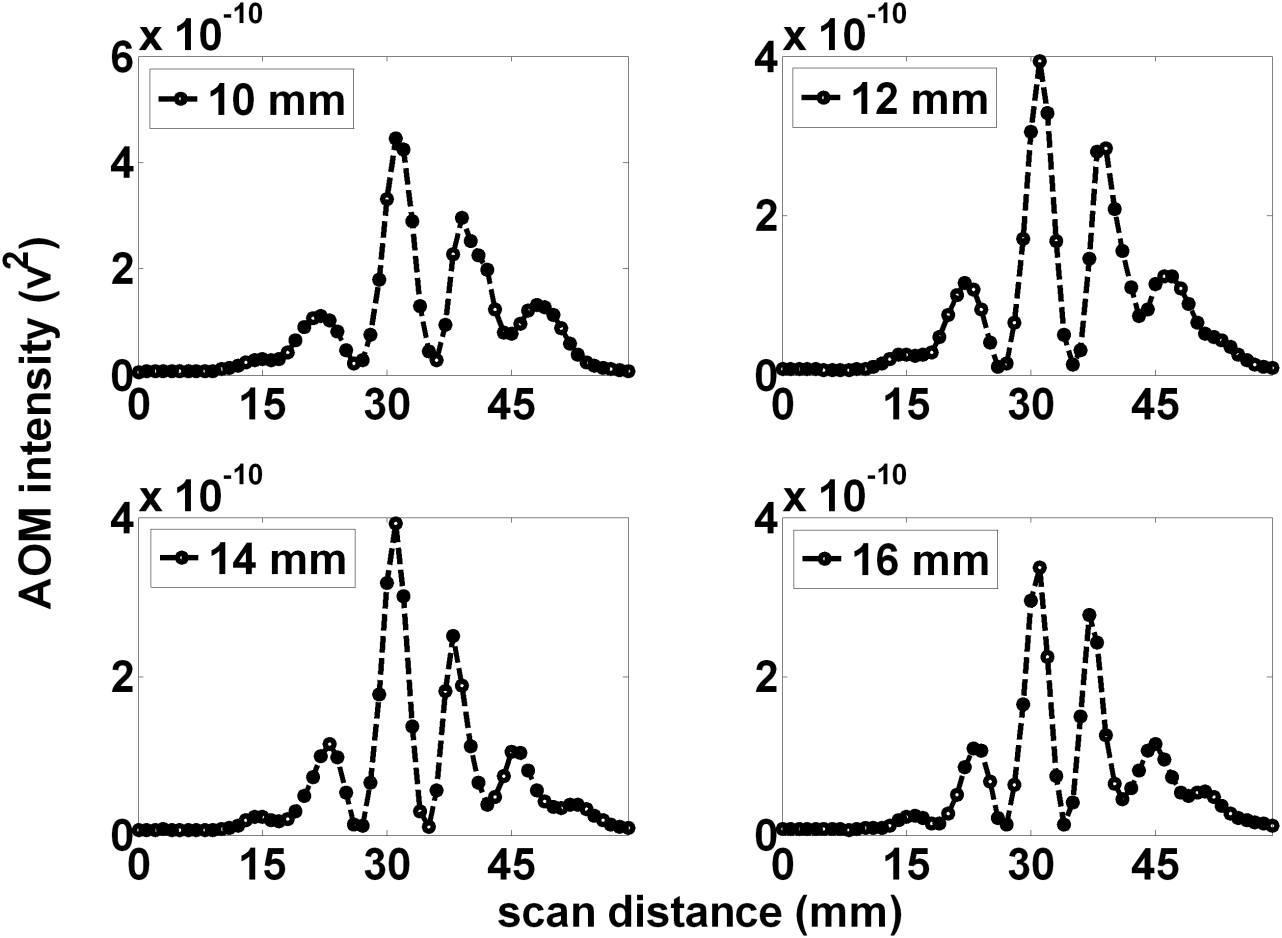
The spatial pattern is suggestive of the alternating regions of optical absorption caused by ultrasound. Increasing the distance between the LED and the ultrasound focal zone causes a narrowing of the overall pattern as well as reduction of the average peak to peak distance. At a projection distance of 113 mm and LED to ultrasound focal zone distance of 10 mm (A), the distant pattern displays alternating peaks with an average peak to peak distance of 8.67 mm. This can be used to calculate an expected 0.77 mm average width of the alternating regions within the ultrasound focal zone. This is precisely the expected value of a half-wavelength of sound in water. As the LED was positioned further from the ultrasound focal zone, (B), (C), and (D), the observed pattern narrowed with additional peaks being added on the fringes of the pattern. In addition, the individual peaks narrow. The pattern is suggestive of the alternating region of optical absorption caused by the ultrasound.

Figure 5 shows the measured projections (for 1 MHz AOM light, within 0.25% volume, whole milk) for various distances (10, 12, 14, and 16 mm) between the LED source and the ultrasound focal zone, with a fixed ultrasound focal zone to PMT distance of 103 mm. As the LED was positioned further away from the focal zone, the peaks in the distant projections undergo shifts that decrease the distance between them, and additional peaks become more clear at the edge of the window. The average distance between peaks decreased as the LED to US focal zone increased (separations of 8.67, 8, 7.5, and 7.33 mm for 10, 12, 14, and 16 mm respectively), consistent with a change in the geometrical magnification of the focal zone by the light source.

**Figure 6:**
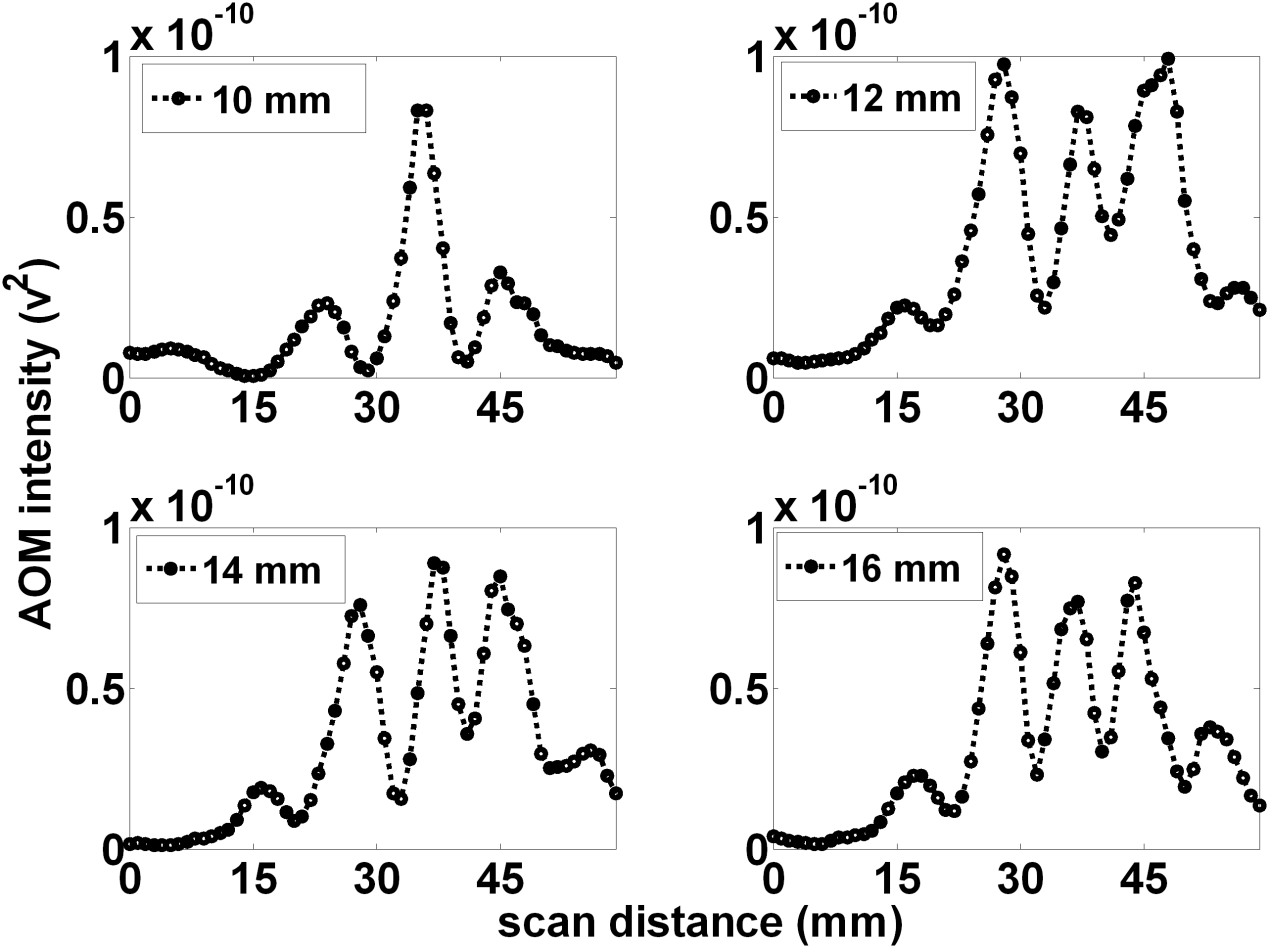
The spatial pattern scales with increased projection magnification. When the projection distance = 140 mm and LED to ultrasound focal zone distance = 10 mm (A), the distant pattern displays alternating peaks with an average peak to peak distance of 10.5 mm. This can be used to calculate an expected 0.75 mm average width of the alternating regions within the ultrasound focal zone. This suggests the pattern scales with expected projection magnification. Similar to figure 5, as the LED was positioned further from the ultrasound focal zone, (B), (C), and (D), the observed pattern narrowed with additional peaks being added on the fringes of the pattern. In addition, the individual peaks narrow.

Figure 6 shows the measured projections (for 1 MHz AOM light, within 0.25% volume, whole milk) for various distances (10, 12, 14, and 16 mm) between the LED source and the ultrasound focal zone but with an increased distance between the ultrasound focal zone and PMT of 130 mm. Similar to the above, the distant projections display small shifts decreasing the distance between the peaks, which also become narrower, with increasing LED to ultrasound focal zone distance (average distance between peaks was 10.5, 10.25, 10, and 8.75 mm for the LED to US focal zone distances of 10, 12, 14, and 16 mm respectively).

**Figure 7:**
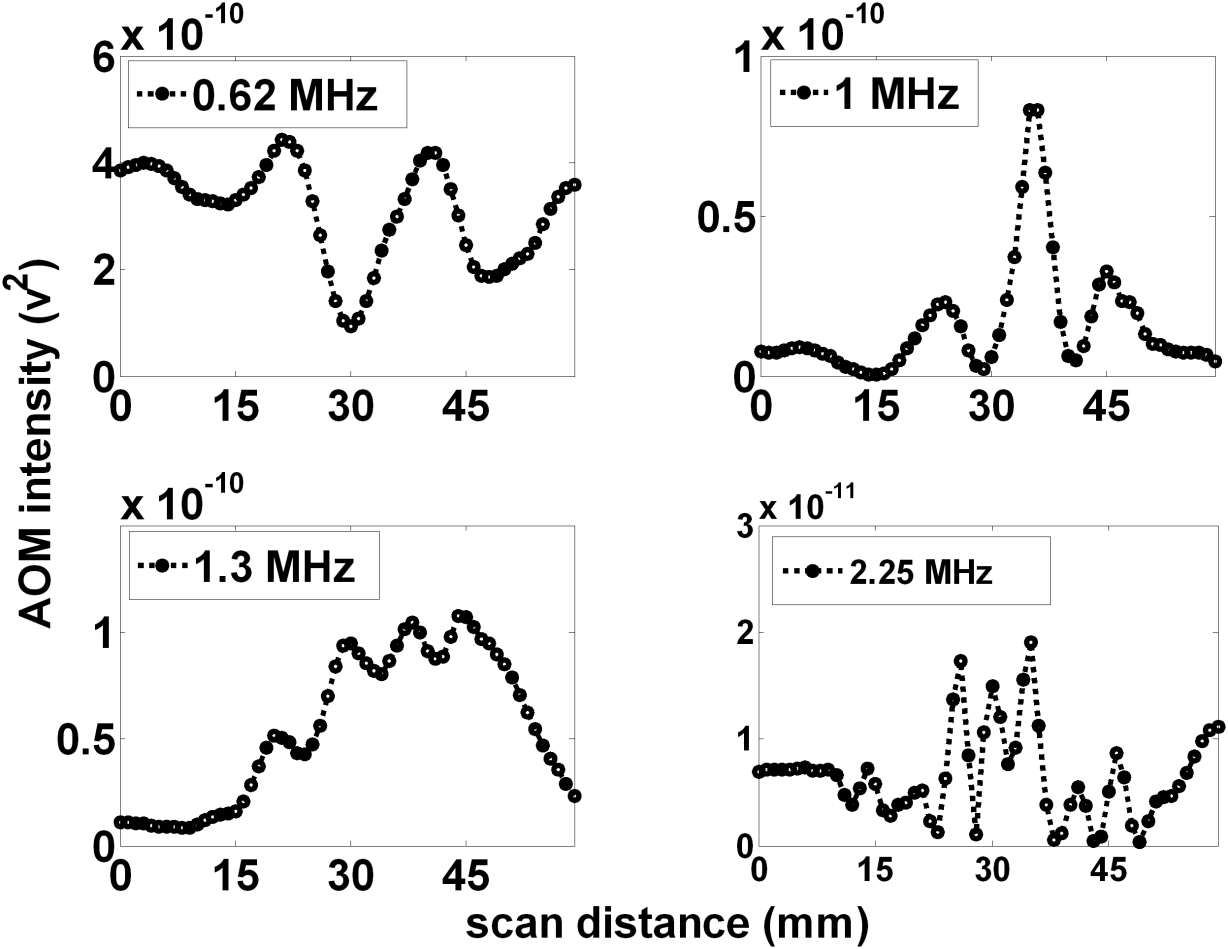
Increasing the applied ultrasound frequency increases the number of projected AOM peaks. The number of observed peaks and their separation within the observed pattern appears to scale with ultrasound frequency, (A), (B), (C), and (D), with 2, 3, 4, and 7 peaks observed for ultrasound frequencies of 0.62, 1.0, 1.3, and 2.25 MHz, respectively.

Figure 7 shows the measured projections (for 1 MHz AOM light, within 0.25% volume, whole milk) for four different ultrasound frequencies (0.62, 1.0, 1.3, and 2.25 MHz) corresponding to wavelengths of 2.4, 1.5, 1.15 and 0.67 mm respectively. The LED to focal zone distance was set at 10 mm and the focal zone to PMT projection distance was set at 130 mm. As the frequency increased, the peaks in the light pattern become closer and more peaks appear within the window. The number of observed peaks was 2, 3, 4, and 7 for US frequencies of 0.62, 1.0, 1.3, and 2.25 MHz respectively.

All experimental data can be found in File S1.

## Discussion

Our results demonstrate that ultrasonic modulation of incoherent light can be measured reliably, a phenomenon often suggested to be too small to observe [27]. Figure 2 shows modulation depths as large as ∼1×10^−8^ can be produced in pure water at readily achievable sound pressures. Furthermore, figures 3, 4, 5, 6, and 7 show that incoherent light can be spatially modulated by sound to generate a pattern consisting of regular maxima and minima across the optical field of view. The pattern scales according to the wavelength and a magnification factor derived from simple geometrical considerations, but as shown below, does not conform to descriptions predicted by earlier theories that invoke wave diffraction and interference effects that are more usually associated with coherent light.

Mechanisms that may produce acoustic modulation of light have been extensively investigated before, and although there are clear explanations for some interactions with coherent light sources, the modulation of incoherent light is less well understood and more variable in practice according to several proposed theories [27], [37]–[39]. Resink [39] has recently reviewed the mechanisms by which photons may be “tagged” by ultrasound, and in experiments using coherent laser excitation and a fluorophore in place of the LED we have observed robust tagging of the laser light causing AO modulation at the ultrasound frequency, as predicted. However, in the current system, the light from the LED is expected to have a coherence length much less than 100 µm [40], and conventional theories of light tagging of such incoherent light do not readily translate to this situation. Moreover, the observed AOM signal pattern clearly arises from the passage of light through alternating regions of optical properties induced by the ultrasound and from our results in pure water (figure 3 bottom) does not depend on the presence of discrete scatterers or induced variations in their number density (which others have suggested may cause AOM signals) [39], [41], [42]. However as the observed pattern were more clearly resolved and quantified because of deeper minima in the turbid media milk experiments, the bulk of the data (figures 4, 5, 6, and 7) are reported as the observations in a 0.25% milk medium. It is known that the sound wave produces periodic variations in density of the medium, which also changes the refractive index of the material [16], including both the real (velocity) and imaginary (absorption) components. From simple acoustic theory, the fractional change in density is expected to be:
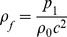
(2)where *p*_1_ is the ultrasound pressure (in Pa), ρ_0_ is the medium density, and *c* is the speed of sound in the medium [43]. At a pressure of 60 kPa the fractional change in the region of maximal compression is approximately 0.0027%. The absorption coefficient of water at 630 nm is 0.319 m^−1^ [44] so traversing a focal zone of dimension 3 mm would lead to variations in absorption (assuming absorption is proportional to density) producing a sinusoidal spatial modulation of the light of maximum amplitude ≈2.4×10^−8^ along the sound beam direction from absorption effects alone. The sound wave also likely produces changes in the light velocity, so some refraction changes may also arise that can cause phase shifts, but the relevance of these for incoherent light is not clear and given the above estimate they do not appear necessary to account for the patterns seen. This predicted spatial modulation of light amplitude is on the same order of magnitude as our observed modulation depth, seen in figure 2 as ∼1×10^−8^, and is easily observed with our experimental set-up. Furthermore, figure 2 shows the AOM signal and the modulation depth scale linearly with the squared ultrasound pressure, suggesting the observed affect is linearly proportional to the ultrasound intensity.

**Figure 8:**
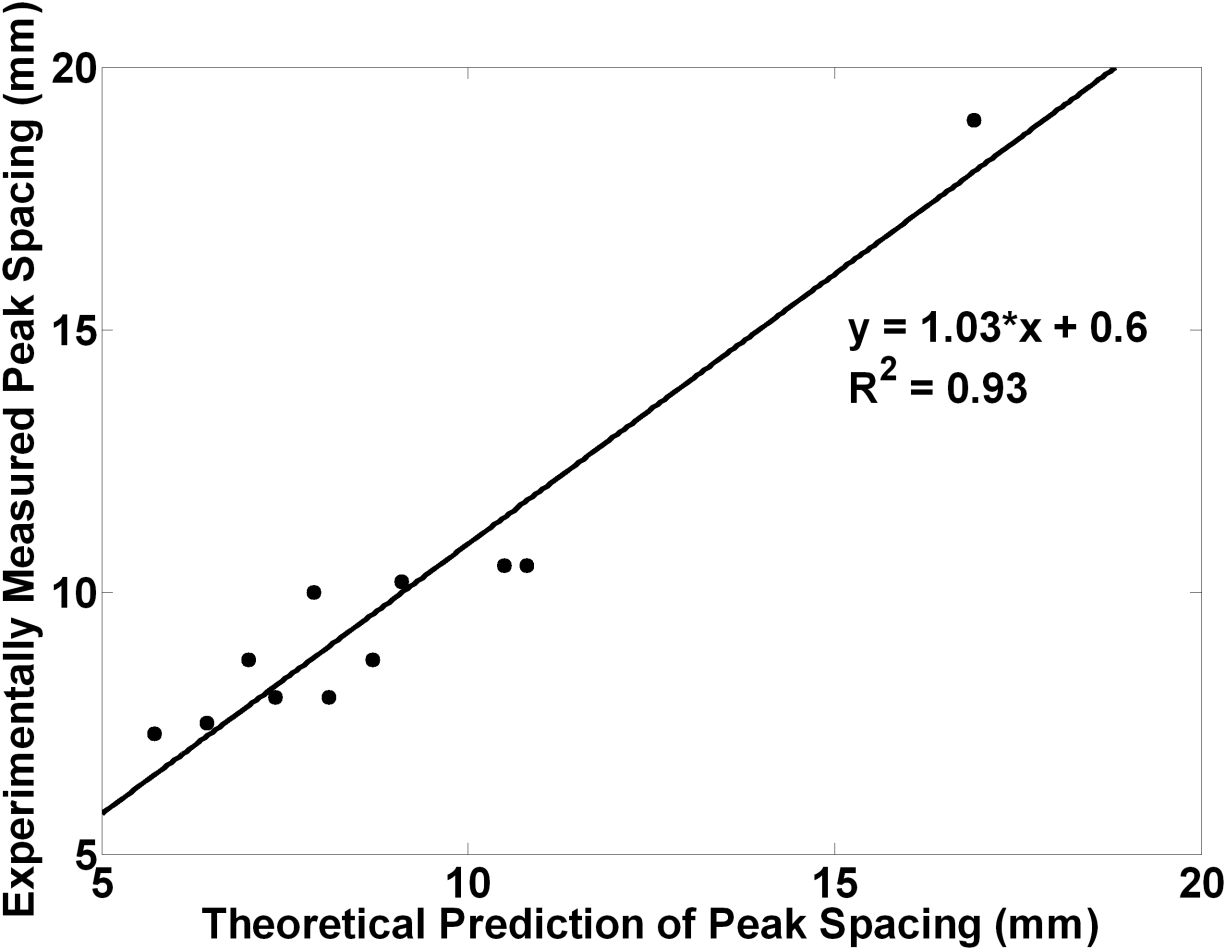
Measured peak separations vs those predicted from simple theory, for different sound frequency and geometry. A linear fit of the observed data agrees with the theory: Measured peak spacing (mm) = 1.03×Predicted spacing+0.6.

**Figure 9:**
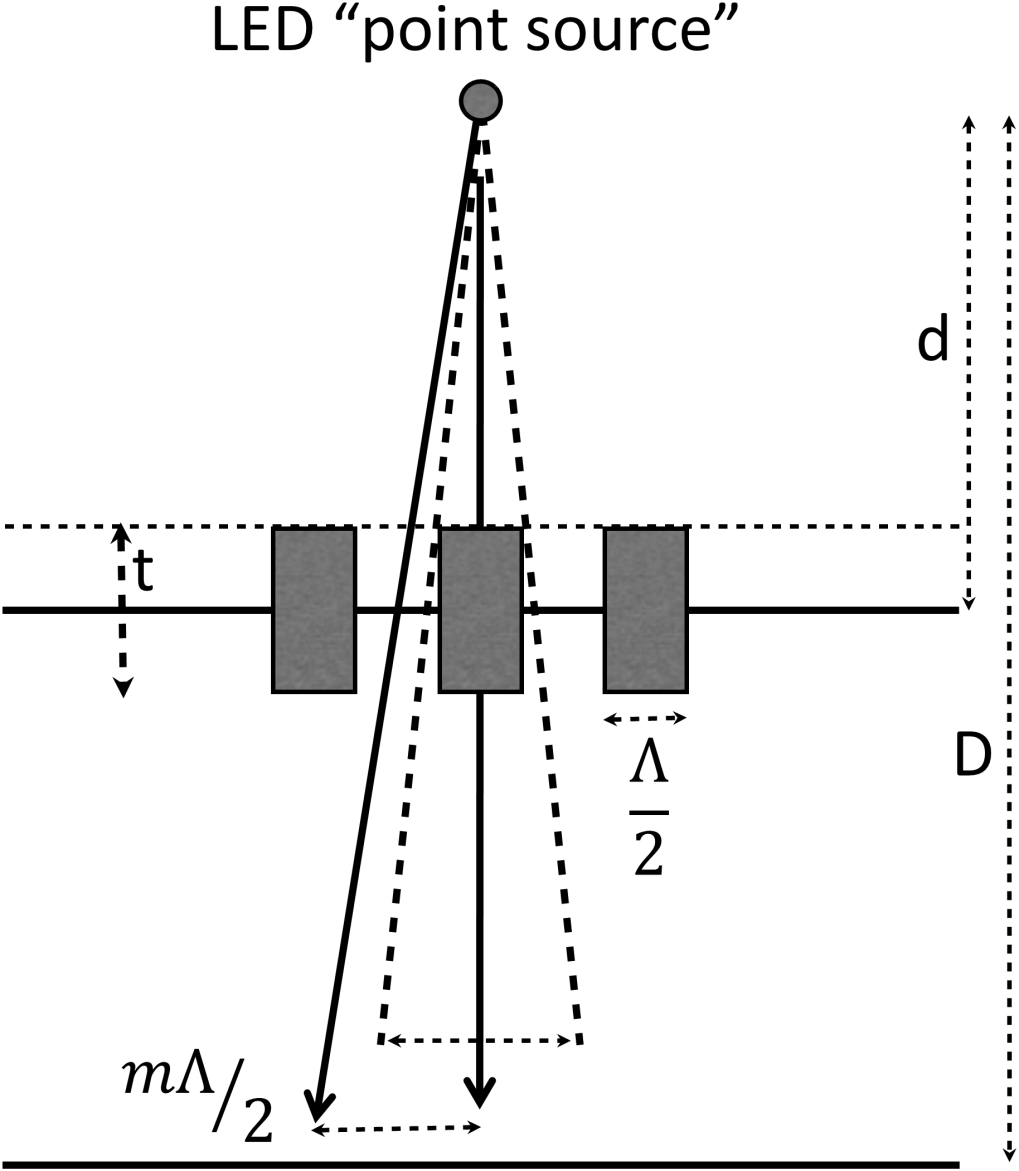
Optical projection of the focal zone. The projection of the focal zone magnifies the spacings and the widths of the peaks with increasing projection distance, and produces unsharp peaks in the spatial pattern. Peak spacings are expected at every ultrasound half-wavelength multiplied by the magnification factor or *mΛ/2*, where *m* is the magnification factor and *Λ* is the wavelength of the ultrasound.

It was observed that the average distance between peaks scaled precisely with the expected geometrical magnification of a simple optical projection of the focal zone when changing the distances from the LED to the focal zone, from the focal zone to the measurement plane, and the wavelength. For simplicity, assume the LED acts as a point source of light. If the distance from the LED to the focal zone axis is d and the distance from the LED to the measurement plane is D then D/d is a geometrical magnification factor m. Distances along the axis of the sound beam become magnified by m at the measurement distance. If the regions of alternating absorption along the axis of the focal zone of the sound beam have a width of a half wavelength 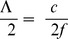 (where *Λ* is the sound wavelength, *f* the frequency, and *c* the speed of sound, taken here to be ≈1500 m.sec^−1^) these become 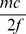 in extent at the light detector. Note that, because we measure the temporally modulated light signal (rather than the mean ambient light level), regions of increased density along the axis show the same modulation as regions of decreased density or rarefaction, so the peaks in modulated light occur every half wavelength rather than every wavelength. As shown in figure 8, the regions of compression and rarefaction also have finite thickness *t* in the direction of light propagation, and a sinusoidal variation along the sound field axis, so their projections are expected to have unsharp edges and may extend over a distance 
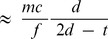
. Thus we predict that alternating peaks of average width up to 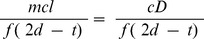 separated by 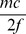, which may reduce the peak to trough modulation. Figure 9 shows the composite data from all the above experiments (varying *m* and *f*), where we have plotted the measured peaks separations versus those predicted by simple theory. The measured separations of the peaks are accurately predicted, and linear regression gives the following relationship.
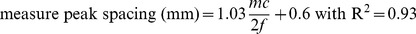



Our data thus appear to suggest that the observed distant patterns are simple optical projections of the absorption pattern produced by the illuminated ultrasonic focal zone. These patterns scale appropriately with the ultrasound frequency and magnification factors. We thus propose that the light intensity propagating through the sound field region is modulated by the ultrasound because of variations in optical absorption alone, and that the distant light pattern depicts the distribution of pressure within the ultrasonic focal zone. This is potentially a new way to image directly the sound fields from ultrasonic transducers.

We emphasize that our experimental findings cannot be explained by previous descriptions of acousto-optic effects. For example, Brillouin first suggested that ultrasound can modulate incident light acting as a sinusoidal grating which optically diffracts incident light into two critical angles [4]. However, Debye and Sears [10] as well as Lucas and Biquard [12] independently proved Brillouin’s predictions inaccurate as they observed multiple orders of diffraction as opposed to the two critical angles Brillouin predicted. Both groups failed to adequately explain the appearance of multiple orders of diffraction but Debye and Sears did suggest that the lack of critical angles was due to the length of the ultrasound interaction with the light. They suggested that the Debye-Sears ratio given by:
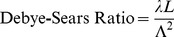
(3)where λ is the optical wavelength, Λ is the acoustic wavelength, and L is the interaction length; should be much larger than 1 for the appearance of the critical angles predicted by Brillouin. This ratio was later refined by Klein and Cook into the Klein-Cook parameter, Q [45], defined as:
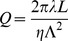
(4)where λ is the optical wavelength, L is the width of the sound beam, η is the index of refraction the medium, and Λ is the acoustic wavelength. When Q>>1, this is currently called the Bragg regime as Bragg was the first to witness this type of diffraction into critical angles using x-ray diffraction in crystals [11]. Despite unsuccessful attempts by Debye and Sears [10], Lucas and Biquard [12]. and Brillouin [46], Raman and Nath provided a series of papers [5]–[9] which explain the appearance of multiple orders with an exact derivation of the relationship between orders. The diffraction regime which causes multiple orders of diffraction is now known as the Raman-Nath regime.

In the Bragg regime, a diffraction pattern consisting of a zero order diffracted beam and a downshifted or upshifted first order diffracted side lobe should arise, while in the Raman-Nath regime a diffraction pattern with multiple diffraction orders projected symmetrically about the zero order is predicted [47]. We can dismiss Bragg diffraction as a possible explanation of our observed results because there are multiple peaks in the observed pattern and the experimental parameters do not meet the Klein-Cook condition [45] of Q»1. Given our set of experimental parameters (λ = 630 nm, L = 3 mm, Λ = 1.5 mm@1 MHz), and assuming an index of refraction of water η = 1.33 [48], the Klein-Cook parameter can be calculated to be Q≈4×10^−9^. Therefore Q«1 and meets the criteria for Raman-Nath diffraction. Within this regime, it is expected that the diffraction pattern will display multiple diffraction orders adjacent to the zero order at angles θ_m_:
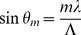
(5)where m is the order of diffraction, λ is the wavelength of the light, and Λ is the wavelength of the acoustic source. Given our experimental values, the value of θ_1_ was calculated to be about 0.02°. Therefore under Raman-Nath theory, the diffraction pattern would generate a first order maximum located at:
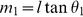
(6)where m_1_ is the distance from the central maximum and l is the distance between the acoustic plane and the observation plane. For our experiment where l = 103 mm, this equates to an expected first order diffraction maximum located at 0.035 mm from the central peak whereas the experimentally observed first order peak location was located at an average 8.67 mm (figure 5, top left): when l = 130 mm the first order diffraction maximum would be expected at 0.045 mm whereas the observed maximum was located at an average 10.5 mm. These discrepancies are so large they cannot be accounted for by Raman-Nath or other diffraction theory.

## Conclusion

We have reported the observation of acoustically modulated incoherent light within optically clear and turbid media which produces a spatial pattern that appears a simple projection of the variation of acoustic pressure within the focal zone of the sound field of an ultrasound transducer. The AOM signal and modulation depth are directly related to the sound intensity. The peaks in the projected light pattern correspond with the expected spacings of density variations and change with the geometrical magnification and wavelength of the sound field. We propose that the ultrasonic waves generate alternating regions of density that produce variations in absorption even in a clear medium such as water. Additional effects of changes in scattering number density or phase shifts and interference caused by variations in the real part of the refractive index do not appear to be necessary to explain these observations. In principle this type of coherent modulation of incoherent light could be used in novel imaging schemes, it may be relevant for the interpretation of some other studies, and may provide a novel way to image complex sound fields directly.

## Supporting Information

File S1 **Final Data.** Includes all data for each figure in 7 separate tables. The tables are Table S1-Figure 2 Data, Table S2-Figure 3 Data, Table S3-Figure 4 Data, Table S4-Figure 5 Data, Table S5-Figure 6 Data, Table S6-Figure 7 Data, and Table S7-Figure 8 Data.
(XLSX)
